# Supplementary material for: Simultaneous estimation of seasonal population density, habitat preference and catchability of wild boars based on camera data and harvest records
Source: R Soc Open Sci. 2020 Aug 19;7(8):200579. doi: 10.1098/rsos.200579 (PMC7481676; doi:10.1098/rsos.200579)
Supplement: Figure S1-S6, Table S1 [file rsos200579supp1.docx]

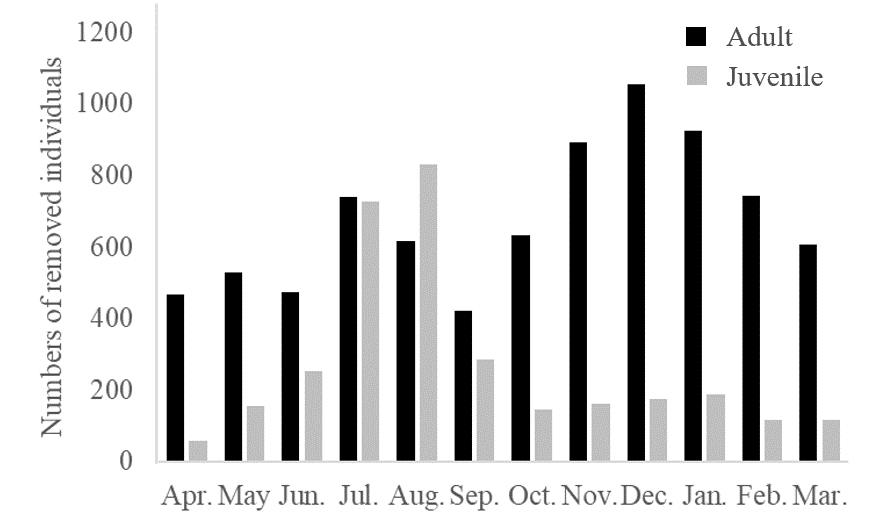


Fig. S1 Numbers of individuals removed by permitted trapping and hunting in 2018 in the whole study area, which are provided by municipal governments.

(a) (b)


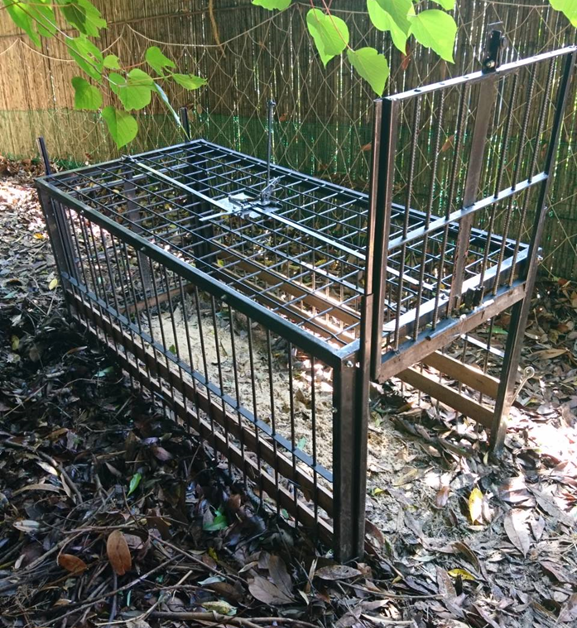

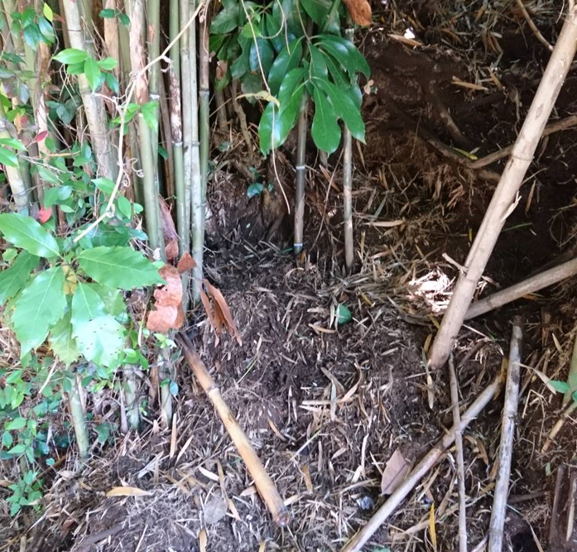


Fig. S2 Two types of traps used for capturing wild boars in the study area.

Box trap is on the left side, and snare traps is on the right side. The capture part of the snare trap is buried underground.


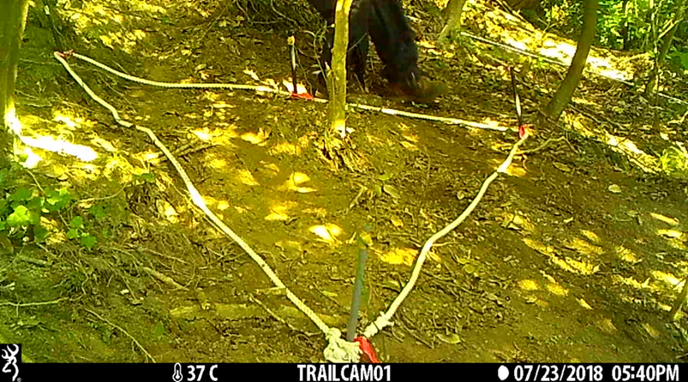


Fig. S3 A photo of the focal area in front of a camera trap, which is used for the REST model. After intial recording of the focal area by the camera, ropes were removed for subsequent monitoring.

| Table S1. Values of WAIC (Watanabe-Akaike widely applicable information criterion, Watanabe 2013) after fitting log-normal distribution with and without random effect at camera stations to the staying time. Minimum values are shown in bold. | | | | | |
| --- | --- | --- | --- | --- | --- |
| No. | Age | Year | Month | With random effect | Without random effect |
| 1 | Adult | 2018 | 6 | 280.0 | **267.3** |
| 2 | Adult | 2018 | 7 | 292.7 | **270.4** |
| 3 | Adult | 2018 | 8 | **259.5** | 264.0 |
| 4 | Adult | 2018 | 9 | 261.1 | **247.7** |
| 5 | Adult | 2018 | 10 | 251.6 | **238.5** |
| 6 | Adult | 2018 | 11 | 318.2 | **293.2** |
| 7 | Adult | 2019 | 12 | 309.5 | **283.1** |
| 8 | Adult | 2019 | 1 | 289.4 | **282.7** |
| 9 | Adult | 2019 | 2 | 298.8 | **289.8** |
| 10 | Adult | 2019 | 3 | **267.9** | 268.5 |
| 11 | Adult | 2019 | 4 | 275.8 | **267.8** |
| 12 | Adult | 2019 | 5 | 286.5 | **276.0** |
| 13 | Juvenile | 2018 | 6 | 259.8 | **256.5** |
| 14 | Juvenile | 2018 | 7 | 297.8 | **283.6** |
| 15 | Juvenile | 2018 | 8 | 279.3 | **263.7** |
| 16 | Juvenile | 2018 | 9 | 272.2 | **271.2** |
| 17 | Juvenile | 2018 | 10 | 103.2 | **99.3** |
| 18 | Juvenile | 2019 | 5 | 115.7 | **112.3** |

(a)


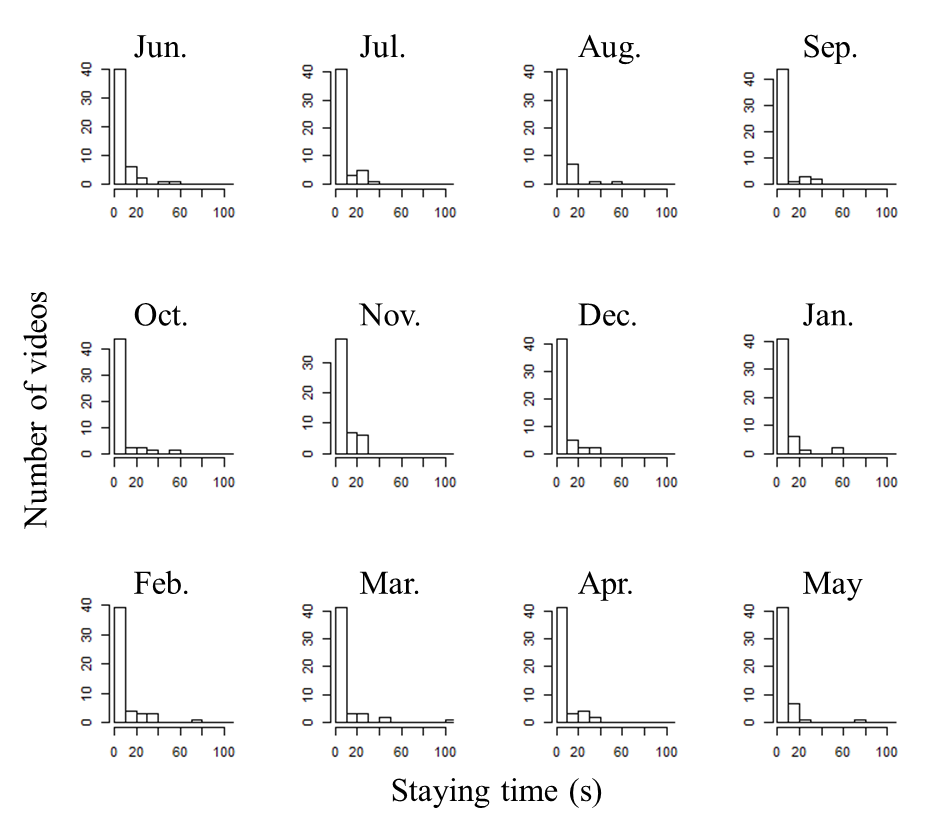


(b)


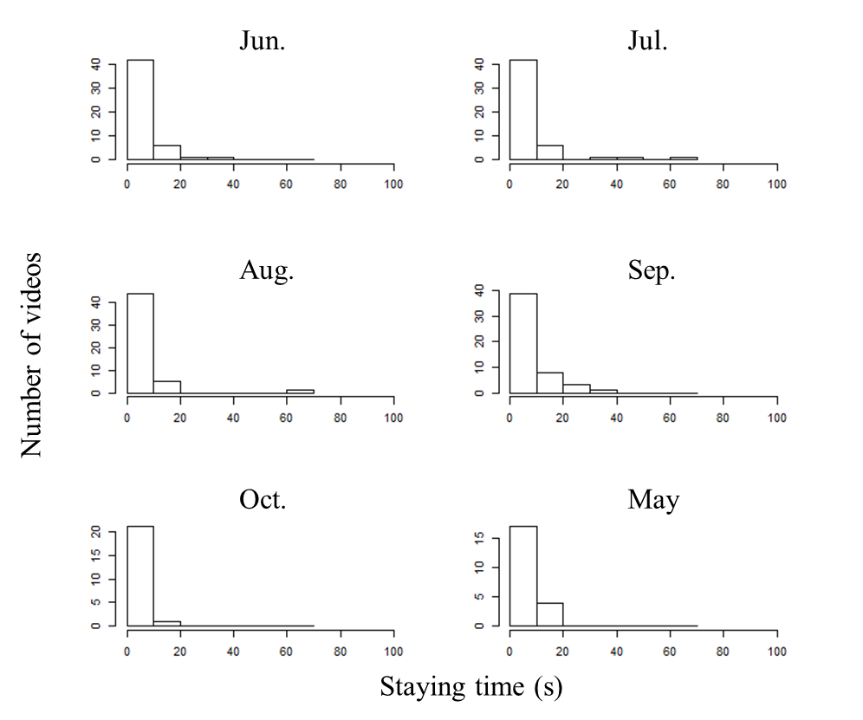


Fig. S4 The histograms of staying time within the focal area in each month ((a): adults, (b): juveniles). Staying time was measured from video recordings at our labolatory.

(a)


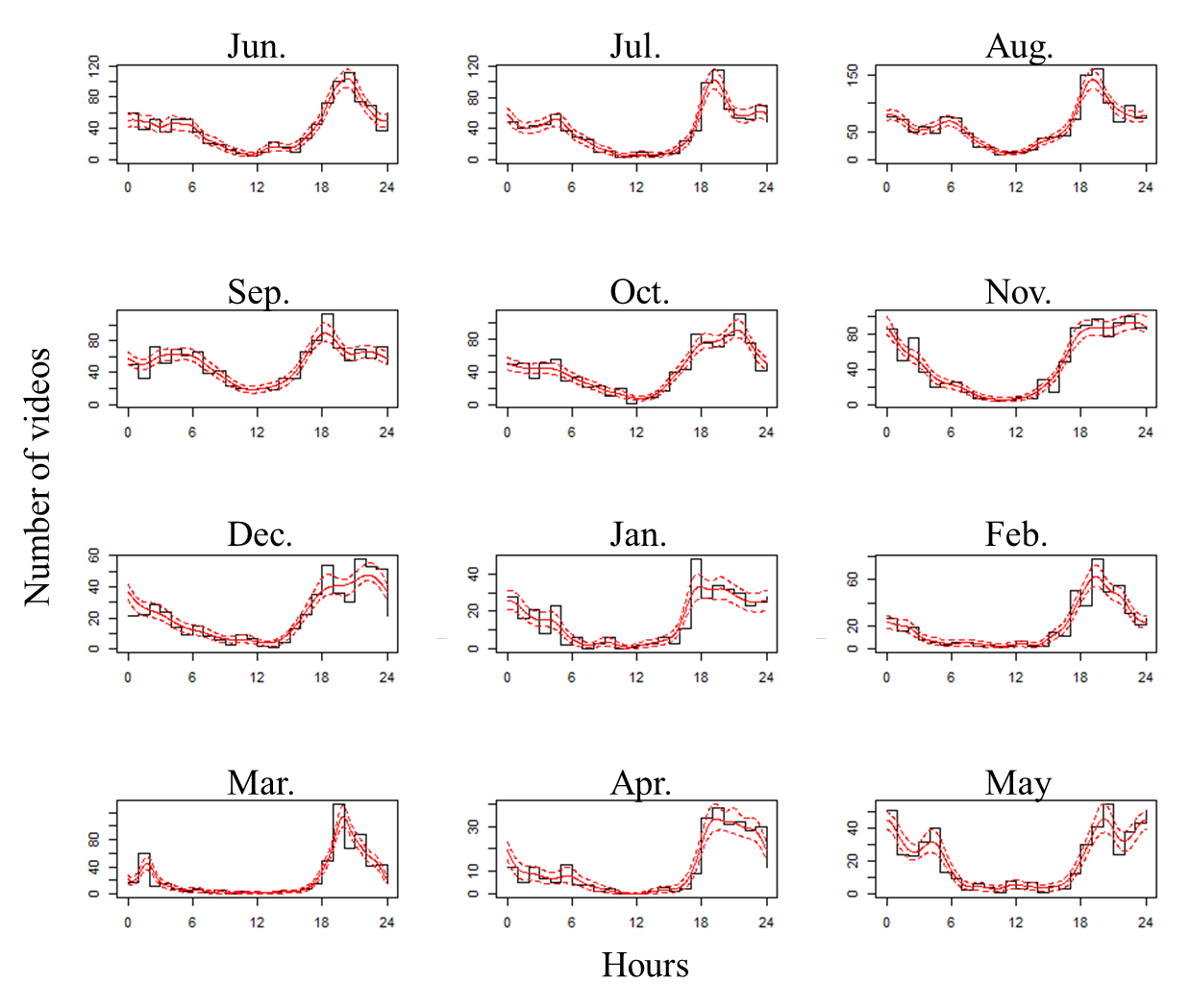
 (b)


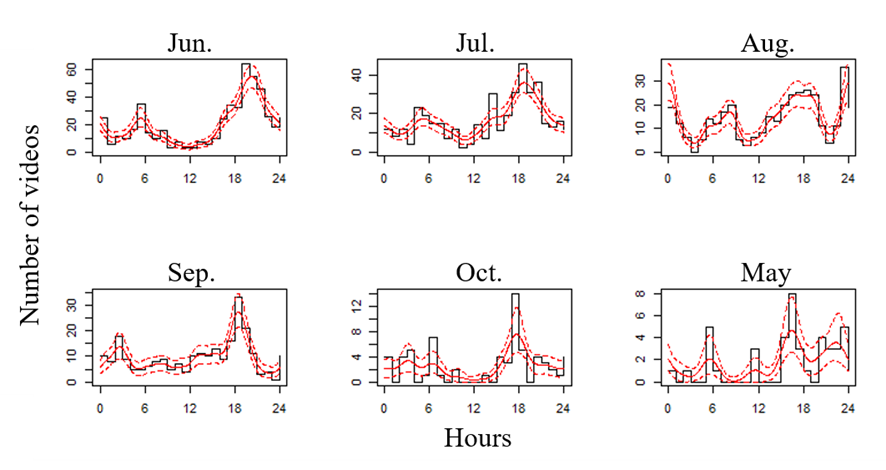


Fig. S5 The Activity proportions for wild boars in each month ((a): adults, (b): juveniles). The activity proportions every 15 minutes were estimated by the method in Rowcliffe et al. (2014). The curve stands for the relative daily activity, estimated by R package “activity” (Rowcliffe 2016). Broken lines represent 95% confidence intervals.

**Reference**

Rowcliffe, J. M., Kays, R., Kranstauber, B., Carbone, C., & Jansen, P. A. (2014). Quantifying levels of animal activity using camera trap data. Methods in Ecology and Evolution, 5(11), 1170-1179.

Rowcliffe (2016). activity: Animal Activity Statistics. R package version 1.1. <https://CRAN.R-project.org/package=activity>


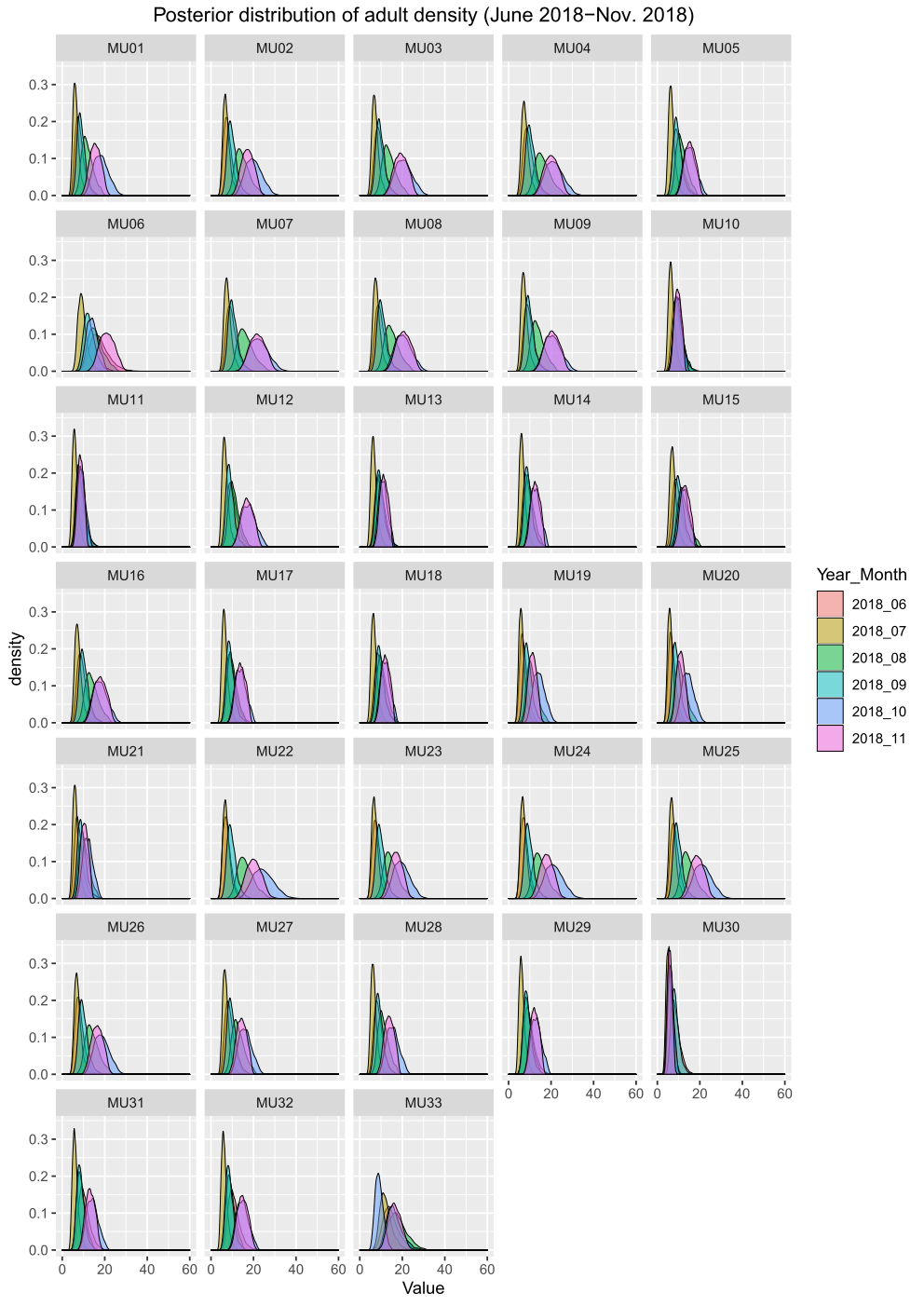


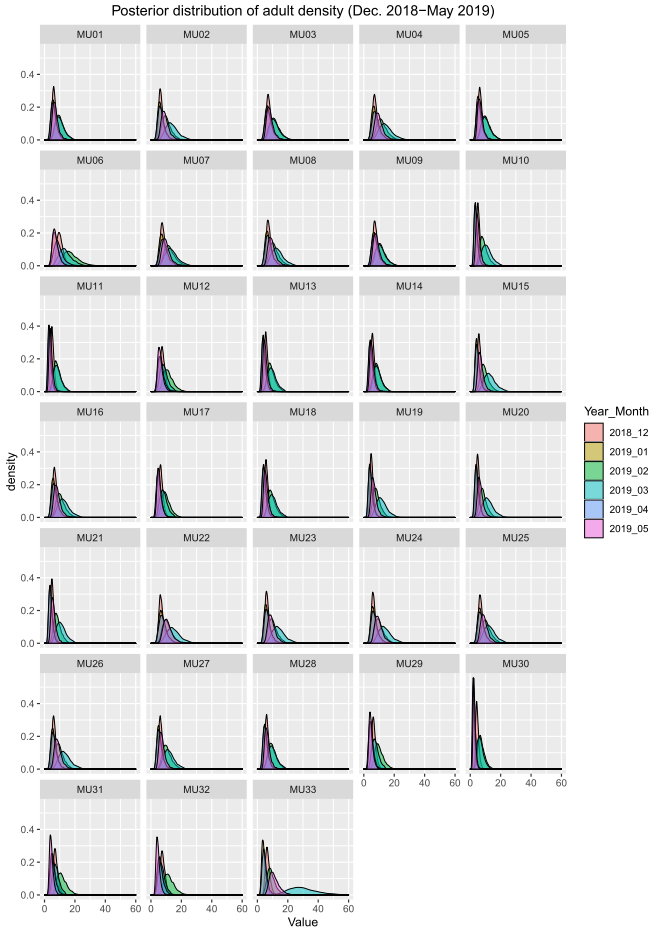


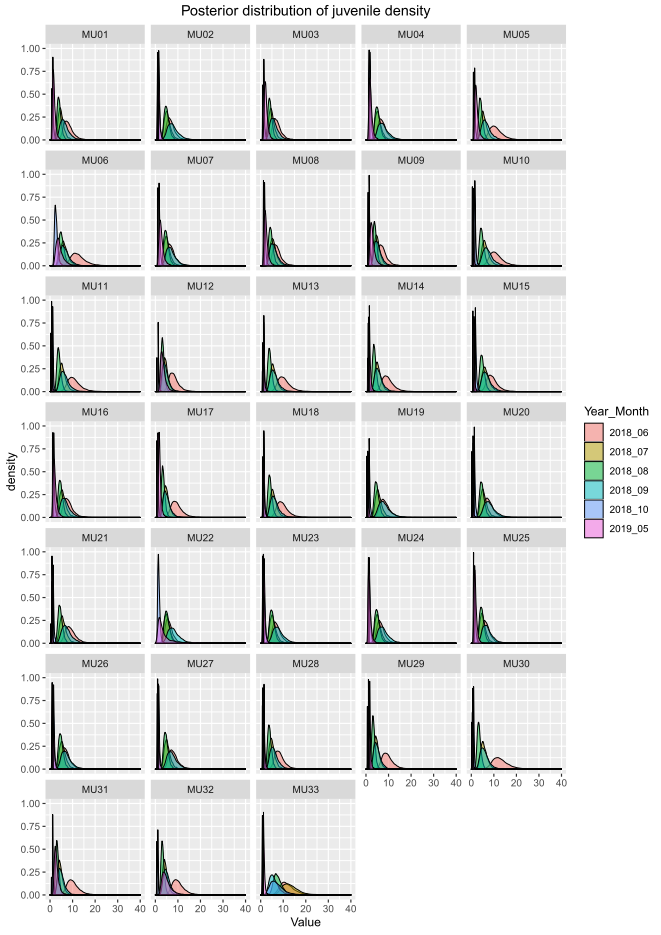


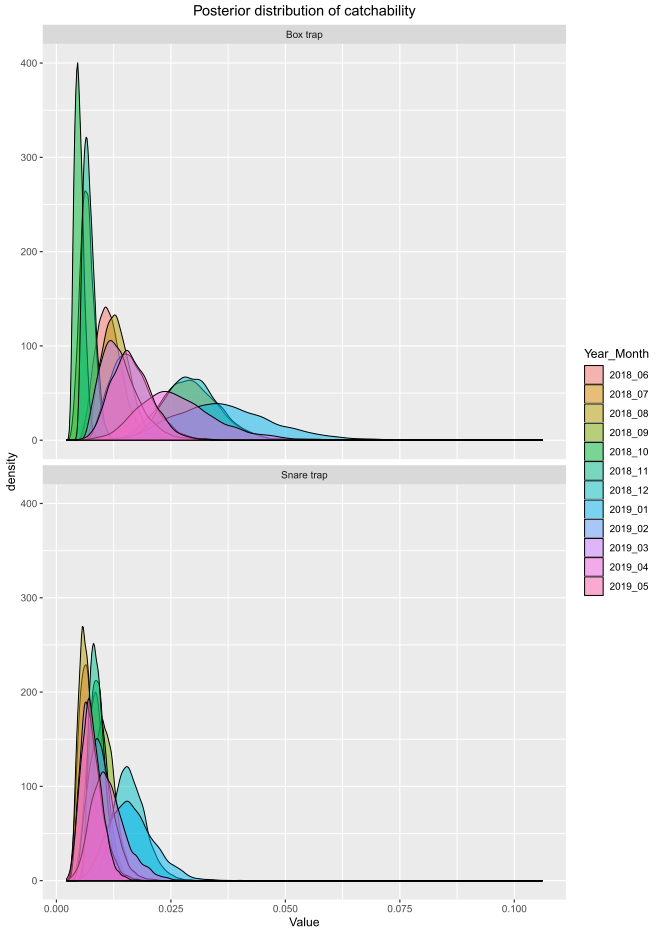


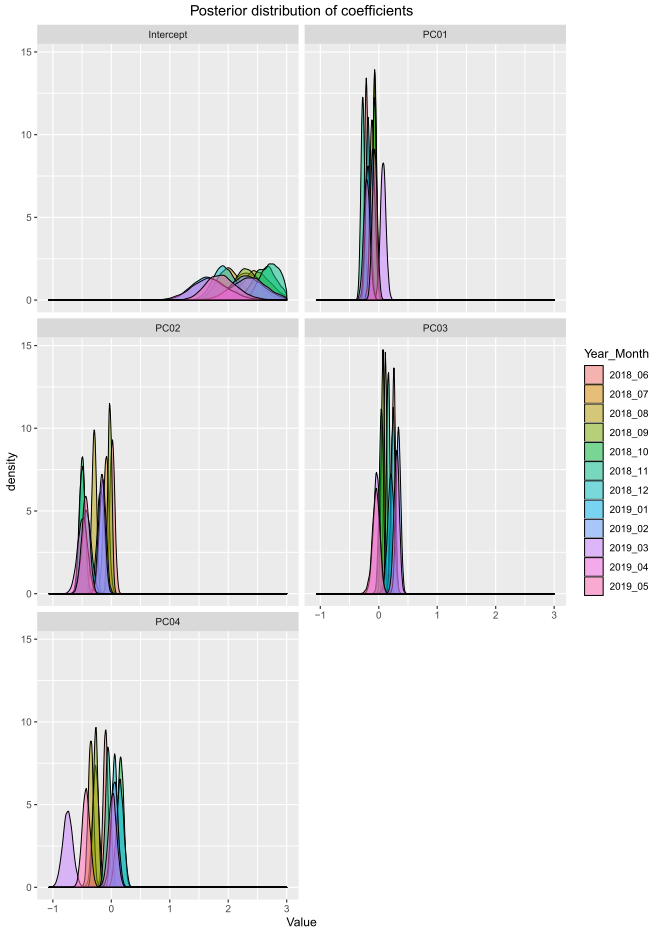


Figure. S6 Posterior distribution of each parameter

The number of MC iterations was 50000, the burn-in was 10000 iterations, and the thinning interval was 20. The number of chains was 3.
